# Impact of Psychosocial and Palliative Care Training on Nurses’ Competences and Care of Patients With Cancer in Cameroon: Protocol for Quasi-Experimental Study

**DOI:** 10.2196/64871

**Published:** 2025-01-03

**Authors:** Nahyeni Bassah, Nicholas Tendongfor, Bachi-Ayukokang Ebob-Anya, Vivian A E Eta, Malika Esembeson, Ndzi Eric Ngah, Salisu Ango Abdul-Rahim

**Affiliations:** 1 Department of Nursing University of Buea Buea Cameroon; 2 Department of Public Health and Hygiene University of Buea Buea Cameroon; 3 Regional Hospital Buea Buea Cameroon; 4 Palliative Care Unit Cameroon Baptist Convention Health Services Mutengene Cameroon; 5 National Radiotherapy Oncology and Nuclear Medicine Centre Korle-bu Teaching Hospital Accra Ghana

**Keywords:** palliative care, psychosocial nursing, oncology nursing, nurses, quality improvement, training, competencies

## Abstract

**Background:**

Cancer is a leading cause of global mortality, accounting for nearly 10 million deaths in 2020. This is projected to increase by more than 60% by 2040, particularly in low- and middle-income countries. Yet, palliative and psychosocial oncology care is very limited in these countries.

**Objective:**

This study describes a protocol for the development, implementation, and evaluation of a psychosocial oncology and palliative care course on Cameroonian practicing nurses’ knowledge, self-perceived competence, and confidence in palliative and psychosocial oncology care provision for patients with cancer.

**Methods:**

A single group pre-posttest design, incorporating both quantitative and qualitative methods will be used. First, a psychosocial oncology and palliative care course for practicing nurses in Cameroon will be developed. This course will then be implemented with 50 practicing nurses purposefully selected from 2 oncology units in the Littoral region and 4 hospitals in the Southwest region of Cameroon. Finally, to assess the impact of the training program we will undertake a pre and posttest survey of nurses’ palliative and psychosocial oncology competences, a pre and post training audit of patients’ nursing records to examine nurses’ practice of palliative and psychosocial oncology care and undertake a critical-incident interview of nurses’ transfer of learning to practice. Descriptive and inferential statistics will be used to analysis quantitative data, while qualitative data will be analyzed using the framework approach.

**Results:**

This study was funded in September 2023. The training program development was initiated in March 2024 and completed in June 2024. Baseline data collection commenced in May 2024 and as of September 2024, we had collected data from 300 patient record. Training implementation is planned for October-December 2024, and post intervention data will be started in October 2024 and continue till April 2025. Data analysis will commence in October 2024 and we aim to publish study findings in peer review journals by November 2025.

**Conclusions:**

This study will improve our understanding of Cameroonian nurses’ palliative and psychosocial oncology competency gaps. It will result in the development of a palliative care and psychosocial oncology course and in the training of 50 nurses in psychosocial oncology and palliative care in Cameroon. This study will inform strategies for future psychosocial oncology and palliative care training initiatives in Cameroon and other low- and middle-income countries.

**International Registered Report Identifier (IRRID):**

DERR1-10.2196/64871

## Introduction

The global need for palliative and psychosocial oncology care continues to grow with the increasing prevalence of life-limiting conditions such as cancer [1,2]. In 2020, there were 19.3 million new cancer diagnoses and close to 10 million cancer-related deaths worldwide. As high as 1.9 million of these deaths occurred in low- and middle-income countries (LMICs) [3]. The number of people requiring palliative care globally has increased significantly in the last 2 decades, from 20 million in 2014 [4] to 56.8 million in 2020 [5]. Unfortunately, LMICs, especially those in sub-Saharan Africa, who have little or no psychosocial oncology and palliative care services harbor up to 80% of this global need [1,6-8]. The limited palliative and psychosocial oncology services in these countries has been associated with barriers such as the lack of competent health care providers with palliative and psychosocial oncology skills, the nonintegration of palliative and psychosocial care in the existing health care system [7,8], the lack of policies to support effective palliative and psychosocial care provision [7,9], and the presence of legislation that limits opioid prescription resulting in poor pain relief, among others [10]. There is therefore a need for strategies to overcome these barriers and facilitate the integration of palliative and psychosocial oncology care into the national health care systems in LMICs [7,11]. Providing educational opportunities to enhance the skills of health care providers in palliative and psychosocial oncology care is one important strategy to ensure that patients have early access to these essential health care services [5,6,12].

Patients and their families who receive a cancer diagnosis experience a high prevalence of pain, emotional, spiritual, and psychosocial distress [9]. The provision of palliative and psychosocial oncology care to these patients and their families will help prevent and relieve these problems [1,6,9] and is cost effective [13]. This is therefore priceless in LMICs where there are limited health care resources, and the cost of care is mostly borne by patients and their families who lack the needed financial resources [13].

With the increasing incidence of cancer in Africa, oncology capacity building opportunities are needed to enhance health care provider’s competences and possibly ensure adequate management of patients and their families [14]. Palliative and psychosocial care are important components of quality cancer care that reduces distress, anxiety, and depression and improves patients’ quality of life and survival [6]. However, it requires close monitoring and presence, which may not be feasible for an already overloaded oncologists [15]. Thus, nurses are in an ideal position to provide palliative and psychosocial care to patients with cancer, particularly in Africa, with huge shortage of oncologists and physicians [16]. Nurse-led palliative and psychosocial have been shown to improve access to palliative and psychosocial care for patients with cancer in resource limited settings [15,17].

### The Need for Palliative and Psychosocial Oncology Care in Cameroon

In Cameroon, there is an upsurge of noncommunicable chronic disease morbidity and mortality [18]. Although cancer is likely underreported, there were approximately 19,564 new cancer diagnoses and 12,798 cancer deaths in Cameroon in 2022 [19]. Similar to most sub-Saharan African countries access to cancer care in Cameroon is limited, although cancer is a leading cause of premature death in the country [20]. There are 3 main cancer centers situated in 2 regions of the country, serving patients from all 10 regions of the national territory [21-23]. The majority of patients visiting these centers present with advanced cancer diagnoses due to delays in access to screening, diagnosis, and treatment [23]. With the limited number of oncology centers and specialists in the country, patients with cancer are often admitted in secondary level hospitals found in each of the 10 regions. Thus, patients with cancer are often cared for by nononcology professionals, particularly nurses. The Cameroon ministry of Public Health has developed a national cancer control plan to increase the number of cancer cases detected and treated early [23]. Thus, the need for strategies to promote early and timely access to psychosocial and palliative care. A study in Cameroon on psychosocial distress and quality of life of patients with cancer [22] found that majority of patients (n=83, 69.2%) presented with clinically significant distress. Financial difficulties, fatigue, transportation issues, and difficulties with work or school were the most reported problems. Up to half of the participants had moderate to severe anxiety and depression symptoms. The quality of life was fair, and there was a statistically significant negative relationship between psychosocial distress and quality of life of patients. These patients however lack access to much needed palliative and psychosocial care.

Palliative care was started in Cameroon in 2003 by the Cameroon Baptist Convention Health Services [24]. The literature reports the lack of a palliative care policy framework in the national cancer control plan [8,23], and limited accessibility to palliative care drugs, especially morphine [25]. These represent a huge gap in access to cancer care and is therefore a significant burden on the population. Training nurses to provide palliative and psychosocial oncology care to patients with cancer in Cameroon can improve access to care, patient experiences and quality of life. A few studies in Cameroon suggested that nurses lack knowledge about palliative care and have negative attitudes toward care of the dying [26,27]. However, there has not been any palliative and psychosocial oncology care training targeting practicing nurses in Cameroon.

### Study Aim

The overall aim of this proposed study is to develop, implement, and evaluate the impact of a psychosocial oncology and palliative care course on Cameroonian practicing nurses’ knowledge, and self-perceived competence and confidence in palliative and psychosocial oncology care provision for patients with cancer, using Kirkpatrick’s framework [28] for training program evaluation. Our specific aims are to develop a psychosocial oncology and palliative care training program for nurses in Cameroon, to implement the training program with 50 nurses who provide care to patients with cancer in selected health care facilities in the Littoral and South-West regions of Cameroon, to assess the impact of the training program on nurses’ psychosocial oncology and palliative care knowledge and self-perceived competence and confidence in psychosocial and palliative care provision for patients with cancer after program completion, to assess the strengths and weaknesses of this training program and identify possible implementation challenges to inform future psychosocial oncology and palliative care curriculum initiatives in Cameroon, and to assess nurses’ transfer of their learning from this training program in the care of patients with cancer and perceived impact on patient outcome.

## Methods

### Design and Methods

A single group pre-posttest intervention design will be used in this study and will incorporate both quantitative and qualitative methods of data collection and analysis. The study will be conducted in 3 phases: course development; course implementation, and course evaluation (Table 1). The Kirkpatrick’s framework for training program evaluation will be used to evaluate the psychosocial oncology and palliative care training program. The Kirkpatrick’s framework is selected for its unique focus on evaluating the outcomes of interventions beyond learners’ satisfaction. It consists of four hierarchical evaluation levels including reaction, learning, behavior, and results. Patients and the public will not be involved in the design, or conduct, or reporting, or dissemination plans of the research.

**Table 1:** Summary of the phases of the study.

Study phase and objective	Activities
Phase 1: Develop a psychosocial oncology and palliative care course for nurses	Engage nurse educators, palliative care experts and oncologist as course development team Develop course through Delphi method Inform course development with content from the psychosocial care competencies framework for nurses in Africa [6], the international psycho-oncology society’s core curriculum [29] and a palliative care course for preregistration nurse training in Cameroon [26]
Phase 2: Course implementation	Recruit nurses who care for patients with cancer in 6 hospitals from 2 Cameroonian regions Deliver the course to 50 nurses over a 4 days duration through theoretical lessons and a practical session
Phase 3: Course evaluation	Undertake a pre- and post-training assessment of nurses’ palliative care knowledge, self-perceived competence and confidence in palliative care provision, and nurses’ psychosocial oncology care competencies Conduct a pretraining audit of nursing records of 300 hospitalized patients with cancer to assess nurses’ baseline practice of palliative and psychosocial oncology care Administer an end of course survey to evaluate the strengths and weaknesses of the course After 6 months of training, undertake a posttraining audit of nursing records of 300 hospitalized patients with cancer nursing records to assess change in nurses’ practice After 6 months of training, conduct critical incident interviews with 18 nurses to assess transfer of their learning to practice

### Settings and Sample

The study will be conducted in 6 health care facilities in the South-West and Littoral regions. This will include 2 oncology units in Douala in the Littoral region and 4 health care facilities in Fako in the South-West region. Patients in the Southwest region of Cameroon who receive a suspected diagnosis of cancer are referred to an oncology center in Douala, the neighboring town in the Littoral region. Thus, they travel long distances to access oncology care. Secondary level hospitals located in the Southwest region provide some consultation by a visiting oncologist. There are few health care providers in the Southwest region with palliative care training [30] and we do not know of any training in psychosocial oncology for nurses in Cameroon. A purposive sample of 50 nurses who work in hospital units that provide care to patients with cancer in the study hospitals from the oncology, medical, surgical, and intensive care units will be recruited, 9 nurses from each study site in the Southwest region and 7 nurses each from the oncology units in the 2 sites in the Littoral region. In a similar study in this setting [31], a power calculation showed that using a *P* value of .05 to determine a statistically significant result, 50 participants were required to give 98% power to find a significant difference in palliative care knowledge. In Cameroon, we have an estimate of 1 nurse per 1000 population [32]. In most secondary and tertiary level hospitals, there are about 10 nurses working in each of the targeted units. Thus, 50 nurses will be selected from an approximate population of about 220 nurses. Nurses who have worked in either the oncology, medical, surgical, or intensive care unit for at least 6 months will included in the study. We consider that 6 months of work in any of these units will allow the nurse to have encountered several patients with cancer and gathered relevant knowledge of their needs from which we can learn during the course and to which they can compare their post training competencies. The nurses have to provide consent and agree to participate during the entire training duration.

### Participant Recruitment Strategy

Permission to conduct the study has been obtained from the directors and supervisors of nursing services of the study hospitals. We are requesting assistance from the general supervisors of nursing services in each study site to identify nurses who meet the inclusion criteria of the study. Interested nurses will register for the training by completing an application form. Selected participants will be contacted to complete the course registration process and consent. All participants will provide consent to participate. Personal identifiers, will only be collected to match pretest and post questionnaires. The questionnaires will be anonymized by coding and removal of any personal identifiers.

### Data Collection

All data collection activities will be undertaken by trained research assistants (RAs), under the supervision of the principal investigators. We will train the RAs on how to use the survey instruments and how to conduct the individual critical incident interviews. We will undertake a mock data collection and role play of interviews with the RAs during the training, to ascertain they can undertake the exercise. The principal investigators will undertake validation checks of the data to ensure that data is precise, accurate and complete.

### Study Activities and Data Collection Methods by Aim

#### Study Aim 1: Develop a Psychosocial Oncology and Palliative Care Training Program

We will develop a psychosocial oncology and palliative care training program for Cameroonian practicing nurses using the psychosocial care competencies framework for nurses in Africa [6], the International Psycho-Oncology Society’s (IPOS) core curriculum [29] and the palliative care course developed for preregistration nurse training in Cameroon [26,27]. At the outset of the study, we will establish a curriculum development committee consisting of 3 members including an oncologists, a palliative care nurse, and psycho-oncologist. The research team will work with the curriculum committee to contextualize the competency framework and IPOS core curriculum for use in the training of nurses in Cameroon. This will be done in collaboration with the psychosocial oncology society of Ghana [7]. We will work virtually via email exchanges, using the Delphi strategy [33] and will organize 2 Zoom (Zoom Video Communications) meetings to finalize and validate the training program.

#### Study Aim 2: Implement the Training With Nurses

The training will be organized at the conference room of participating hospitals to facilitate nurses’ participation. Thus, 6 training sessions will be implemented with participants in the 6 study sites. Each training session will last 4 days. Day 1 and 2 will entail classroom training sessions; day 3 will be clinical case studies and scenarios through role playing under the supervision of a palliative care nurse, oncologist, and clinical psychologist and day 4 will be experience sharing, action plan development, and training evaluation. The classroom sessions will include interactive lectures assisted by PowerPoint (Microsoft) presentations, pictures and videos, presentation of case studies, sharing of personal experiences and group discussions of concepts and experiences. An international facilitator will be invited to take part in the training and give an opportunity for others to present virtually during the training. For the clinical case studies and scenarios, participants will be shared in groups of 5 to work on different clinical case studies and scenarios followed by sharing feedback with the entire team. A similar approach has been used and shown to be an effective strategy in the training of nurses in psychosocial oncology [34]. Participants will be provided with printed copies of the palliative care toolkit by Lavy and Woodridge [35]. In addition, they will be provided with links to online resources including the free online resources by IPOS.

#### Aim 3: Assess the Impact of the Training Program on Nurses’ Psychosocial Oncology and Palliative Care Competences

A pretest and posttest survey will be used to assess the impact of the course on nurses’ psychosocial oncology and palliative care knowledge and self-perceived competence and confidence in psychosocial and palliative care provision. An advantage of a pretest and posttest assessment is that it can be used to enhance understanding of what change, particularly in factual knowledge or skill sets that could be credited to a training program. All 50 study participants will receive a paper-based pretest evaluation to determine their baseline psychosocial oncology and palliative care knowledge, and self-perceived competence and confidence in psychosocial and palliative care provision. The paper-based assessment will take place at the hospital on the first day of training. Posttest data will be collected on the last day of the training. A questionnaire has been collated and comprises of 3 subscales to collect the pretest and posttest data. These subscales include a researcher developed demographic information subscale, the Palliative Care Quiz for Nursing [36], Perceived Palliative Care Self-efficacy Questionnaire [37], and the psychosocial oncology knowledge subscales [34]. The demographic questionnaire will collect information on participants’ characteristics including nurses’ qualification, hospital and unit of work, gender, religious affiliation, and previous education about palliative care and psychosocial oncology. The validated Palliative Care Quiz for Nursing will be used to assess change in nurses’ palliative care knowledge, the Perceived Palliative Care Self-efficacy Questionnaire will be used to assess self-perceived competence and confidence in palliative care provision. These instruments have adequate psychometric property and have been used to assess student nurses’ palliative care competencies in the study setting. For the assessment of psychosocial oncology competences, we have adapted the instruments used in the study by Mahendran et al [34] in Singapore.

#### Aim 4: Assess the Strengths and Weaknesses of This Training Program

A course evaluation survey will be administered to participants to explore their experiences of the course, their perspective of its strengths and weaknesses, and their plans for transfer of their knowledge and skills from this course to practice, in the care of patients with cancer. This will include both close-ended and open-ended questions to give nurses the opportunity to expand on their responses and provide all relevant information as they deem necessary. This survey instrument is under development by the team and will be administered by research assistants on the last day of each training session.

#### Aim 5: Assess Nurses’ Transfer of Their Learning From This Training in the Care of Patients With Cancer

Individual critical incident in-depth interviews will be conducted with nurses to explore the transfer of their learning to practice, its perceived benefits to patients and implementation challenges. A random sampling technique will be used to invite 3 nurses from each study site for this interview. Thus, a total of 18 nurses will be interviewed. These interviews will be organized at month 6 following completion of the training for each hospital. An interview guide will be used and it is envisaged that the interviews will last for 1 hour and will be tape recorded. In addition, a pre- and posttraining audit of patient files will be carried out to assess the number of patients with cancer that nurses assessed for psychosocial distress and coping, depression, anxiety, and palliative care, the number they plan psychosocial nursing and palliative care interventions (cognitive behavioral therapy) for and the number that nurses referred or suggest referral to specialist mental health and palliative care services or psycho-oncologists. The pretraining audit will be conducted at baseline, using a checklist. We will select files of the 50 most recently discharged patients with cancer or patients who died during hospitalization from each study site. Thus, a total of 300 hospital files will be studied from the 6 sites. Six months after training implementation in each site, we will undertake the posttraining audit. We will randomly select 50 files of patients with cancer currently receiving or who received nursing care from trained nurses. In total we will study 600 files, 300 from baseline and 300 post training.

### Data Analysis

We will evaluate changes in mean scores from pretest to posttest to assess the impact of the course on nurses’ psychosocial oncology and palliative care knowledge, and self-perceived competence and confidence in psychosocial oncology and palliative care provision. A paired *t* test, will be used to calculate the change in nurses’ psychosocial oncology and palliative care knowledge from pretest scores to posttest scores, and to examine the significance of this change. The between and within group variations of the pretest and the posttest scores in psychosocial oncology and palliative care knowledge before and after the training program will be assessed using ANOVA. The McNemars test will be used to assess changes from the pretest to the posttest in nurses’ self-perceived competence and confidence in psychosocial and palliative care provision. Descriptive statistics (close ended responses) and content analysis strategy (open-ended responses) will be used to analyze data on nurses’ evaluation of the benefits of the course on their psychosocial oncology and palliative care competencies, their experiences of the course and its strengths and weaknesses, and how they were able to use them in practice.

A framework analysis [38] will be used to analyze critical incidents where nurses implemented their learning in practice and their perspective of how that benefited the patients with cancer in their care. Themes will be used to describe these experiences. Finally, a content analysis of the change in nurses’ practice in terms of the number and content of patients’ assessment for psychosocial distress, depression, anxiety, and palliative care, the number of patients with psychosocial and palliative care nursing care plans and the number that were referred to specialist mental health or palliative care services by nurses before and after the training. All project team members, RAs, and program coordinator will be involved in data collection and analysis.

### Ethical Considerations

Ethical approval for this study has been obtained through the University of Buea institutional review board (Ref-2023/2165-10/UB/SG/IRB/FHS). Participants will receive an information sheet regarding the study and will provide written consent for the study. Participation in the study will be voluntary and participants will also have the freedom to leave the study at any time without giving a reason to the study team. Data will be anonymized to ensure and no identifiable information will be stored. No compensation will be provided to research participants.

## Results

This study was funded in September 2023. The training program development was initiated in March 2024 and completed in June 2024. We engaged 12 cancer care, psychosocial oncology and palliative care experts, and nurse educators in Cameroon (n=7), the United Kingdom (n=2), Ghana (n=1), Indonesia (n=1), and the United States (n=1) in a 3-round Delphi process for the development of a bespoke palliative care and psychosocial oncology training program. As of August 30, 2024, we had obtained administrative authorization from all participating hospitals. The collection of baseline data on nurses’ practice of psychosocial oncology and palliative care as documented in the nursing records of patients with cancer was commenced in May 2024. As of September 19, 2024, we had completed an audit of the records of 300 hospitalized patients with cancer from all study sites. The implementation of the training program is planned for October-December 2024. Following course implementation, posttest data will be started in October 2024 and continue till April 2025. Data analysis will commence in October 2024 and will continue until June 2025. We aim to present study findings at national and international palliative care and psychosocial oncology conferences and publish papers in peer review journals by November 2025.

## Discussion

### Expected Project Outcomes

This protocol provides details of the steps for the development, implementation and assessment of the impact of a psychosocial oncology, and palliative care training program on Cameroonian nurses’ palliative care and psychosocial oncology knowledge, self-perceived competencies, and practice. In this study, we are developing a palliative and psychosocial oncology course for nurses in Cameroon. We will pilot it with 50 nurses working in 6 hospitals in the southwest and littoral regions of Cameroon. We envisage that this training program will improve participating nurses’ palliative care knowledge and self-perceived competence and confidence in psychosocial and palliative care provision for patients with cancer. The implementation of this training program will enhance our understanding of the components and strategies for an effective palliative care and psychosocial oncology training program for nurses in Cameroon. We also hope that study findings will support future psychosocial oncology and palliative care training initiatives in Cameroon and similar contexts.

The field of psychosocial oncology is still developing in Africa [39] and therefore, initiatives to enhance health care providers’ competences in this practice area are crucial especially in sub-Saharan Africa. Given the limited numbers of oncologist, psycho-oncologist, mental health experts and palliative care physicians and nurses in Cameroon, it is important to train registered nurses, who make the most of the health care workforce in Cameroon to provide palliative and psychosocial oncology care to patients with cancer and their families. This project builds on previously successful educational initiatives in palliative care for nursing students in Cameroon to enhance care of patients with life-limiting illnesses including patients with cancer [26,27]. Training nurses in palliative care and psychosocial oncology in other contexts have yielded positive impacts in terms of improvements in their knowledge and skills in palliative and psychosocial oncology care provision, with positive patient outcomes [15,34,40,41]. Similar outcomes are anticipated for this training in Cameroon.

Similar to this study, most studies have used both validated instruments such as the Palliative Care Quiz for nurses, and nonvalidated self-reported tools to evaluate the effective of training programs [15,40,41]. However, the use of self-reported tools is limited, with the possibility of recall bias, and social desirability bias [42]. Participating nurses may not recall past practices and may also tend to please the researcher and answer questions in ways they consider right rather than giving a true self-assessment of their competencies. Furthermore, nurses could overrate or underrate their competencies, resulting in a response shift bias [43].

The Kirkpatrick’s model of training program evaluation used in this study provides us with a logical structure and process to measure participants’ learning, satisfaction and transfer of learning to practice. It will provide us with an understanding of the specific areas for improvement within this training program, based on its strengths and weakness, thus informing program enhancement [44]. We aim to disseminate our study’s findings in a peer reviewed journal and local and international conferences. In addition, during program implementation we will engagement with general supervisors of nursing services, nurse educators, and other health care professions education stakeholders in Cameroon. This has the potential to inform nursing education reforms and drive the long-term goal of curriculum revision to include palliative care and psychosocial oncology in the training programs of health professionals in Cameroon.

## Abbreviations

IPOS: International Psycho-Oncology Society
LMIC: low- and middle-income country
RA: research assistant

## Data Availability

The datasets generated during and/or analyzed during this study are available from the corresponding author on reasonable request.
